# The end of the genetic paradigm of cancer

**DOI:** 10.1371/journal.pbio.3003052

**Published:** 2025-03-18

**Authors:** Sui Huang, Ana M. Soto, Carlos Sonnenschein

**Affiliations:** 1 Institute for Systems Biology, Seattle, Washington, United States of America; 2 Tufts University School of Medicine, Immunology, Boston, Massachusetts, United States of America; 3 Centre Cavaillès, Ecole Normale Superieure, Paris, France

## Abstract

Genome sequencing of cancer and normal tissues, alongside single-cell transcriptomics, continues to produce findings that challenge the idea that cancer is a ‘genetic disease’, as posited by the somatic mutation theory (SMT). In this prevailing paradigm, tumorigenesis is caused by cancer-driving somatic mutations and clonal expansion. However, results from tumor sequencing, motivated by the genetic paradigm itself, create apparent ‘paradoxes’ that are not conducive to a pure SMT. But beyond genetic causation, the new results lend credence to old ideas from organismal biology. To resolve inconsistencies between the genetic paradigm of cancer and biological reality, we must complement deep sequencing with deep thinking: embrace formal theory and historicity of biological entities, and (re)consider non-genetic plasticity of cells and tissues. In this Essay, we discuss the concepts of cell state dynamics and tissue fields that emerge from the collective action of genes and of cells in their morphogenetic context, respectively, and how they help explain inconsistencies in the data in the context of SMT.

## Abstract 2

Genome sequencing results and single-cell transcriptomics continue to produce findings that challenge the idea that cancer is purely a ‘genetic disease’. This Essay delves into cancer omics data that questions the somatic mutation theory (SMT), and presents theories based on gene regulatory networks and tissue fields that help explain inconsistencies in the data.

## Introduction

It is said that the wise only believe in what they can see, and the fools only see what they can believe in. The latter attitude cements paradigms, and paradigms are amplified by any new-looking glass that puts one’s way of seeing the world on steroids. In cancer research, such a self-fulfilling prophecy has been fueled by next-generation DNA sequencing. The ease of access to sequencing has stimulated the unfettered quest for genetic alterations in tumor cells that would explain cancer. Since the 1970s, the elevation of the concept of ‘cancer as a genetic disease’ [1–4] has been driven by a number of factors, including discoveries of cancer-associated mutations, narrowly gene-engineered mouse models, funding policies that follow crowd thinking, and the promise of targeted therapy. The propensity of the human mind to cling to simple, mechanistically plausible explanations further promoted narratives of oncogenic ‘driver mutations’ [5–7]. The somatic mutation theory (SMT) and its tacit claim to represent the “truth” on the origin of cancer is concisely articulated in this quotation by M. Stratton and L. Alexandrov from a decade ago:

“*All cancers originate from a single cell that starts to behave abnormally due to the acquired somatic mutations in its genome*” (2014). [8]

This quotation dates back to 2014—and we will present an updated 2024 version later. However, during the past decade, widespread acceptance of the SMT has driven the use of next-generation sequencing. With lowering costs of genome analysis, the genetic paradigm led to ‘precision oncology’; but targeting driver mutations has invariably met the challenge of relapse of treatment-resistant cancer, too fast and in far more aggressive form [9–13] than can be explained by selection for new mutants. Knowing the mutational profile of a patient’s tumor genome has yielded little benefit in ‘personalized/precision’ cancer care [7,9,14,15].

Ironically, over the past years, the same sequencing technology has exposed new cracks in the edifice of the cancer genetics paradigm. Table 1 summarizes the most salient findings that challenge the paradigm. Some sequencing results are compatible with the postulate of oncogenic mutations, but many inconvenient findings are overlooked for lack of critical discourse, resulting in an unbalanced view. Thus, an open discussion of the growing sequencing data that are contradictory to the genetic paradigm is due. The (apparent) paradox goes both ways: many cancers harbor no consistent driver mutations, while canonical oncogenic mutations are found in tissues that remain free of cancer [16] (see Table 1 for references). Paradoxes, according to Nils Bohr, are of course central for progress [17]—if properly recognized.

**Table 1:** ‘Paradoxical’ findings regarding the genetic paradigm of cancer uncovered by sequencing technology.

	Observation	Details/ Remarks	References
**1**	Some tumor genomes display very few or no recurrent genomic mutations despite a recurrent, characteristically abnormal phenotype.	Examples for cancers that lack (consistent) oncogenic mutations include rhabdomyosarcoma and ependymoma.	[16,18,19]
		Conversely, mutational signatures known to be associated with exposure to chemical carcinogens can be indistinguishable from that of sporadic cases of the same cancer.	[20]
		Along the same line, almost every gene in the genome has been associated with cancer.	[21]
**2**	Genomic DNA of normal tissues contain numerous mutations, including those considered oncogenic.	That normal tissues can ‘tolerate’ mutations and stay normal without transforming has long been noted. This is evident in the ubiquitous accumulation of somatic mutations in non-cancerous tissues in individuals with inherited cancer syndromes that affect DNA repair.	[22–26] [27] [28]
**3**	Indolent and potentially premalignant lesions consistently contain driver mutations, yet rarely progress to cancer.	In naevi (moles on the skin), Barrett’s esophagus, or ductal carcinoma in situ (DCIS), ‘oncogenic driver’ mutations, such as in BRAF, TP53 or PI3K, etc., are abundantly found and seem to confer certain degree of clonal dominance in these non-cancerous tissues. Only a small fraction (e.g., ~ 1% for Barrett’s esophagus and 20% for DCIS) ever progress to cancer that intriguingly is often clonally unrelated to the early lesion, suggesting field cancerization (see point 7).	[29–33]
**4**	Ionizing radiation of cells does not trigger clonal expansion of mutated cells—instead, multi-clonal growth ensues after several cell generations.	Instead of the assumed expansion of a single mutated tumorigenic clone after genotoxic irradiation, genetic alterations appear only several cell generations later and are multi-clonal. Thus, ionizing irradiation causes neoplasia via cytoplasmic or microenvironmental events, such as cell stress (replication stress) or tissue injury and inflammation that may result in genome instability (see point 10).	[34]
**5**	Tumor cells carrying oncogenic mutations are not necessarily under positive selection—but they are usually too rare to drive progression via selection for new malignant phenotypes.	A central tenet of SMT is that cells carrying the tumor-driving mutation(s) should be under positive selection and take over the tumor cell population (selective sweep and genetic fixation). Such events are barely seen. However, neutral drift can extensively propagate non-advantageous mutations by chance, creating the semblance of enrichment in genetically heterogeneous cell populations. Marginal positive selection of a mutated gene (as detected by codon analysis) does occur, albeit rarely. Yet for the logic of precision treatment that seeks to eradicate the tumor, genetic fixation is required; otherwise, residual disease is guaranteed.	[35–40]
		The most plausible cases of positive selection are point mutations in proteins that interfere with target-selective inhibitor drugs, thereby conferring drug resistance.	[41]
**6**	Genetic (clonal) heterogeneity and reconstructed lineages of cancer cells reveal complex subclone kinetics with branching, parallel and non-monotonic patterns, defying stepwise (linear) clonal selection.	Long noticed in deep or multi-site sequencing of tumor genomes is the coexistence of hundreds of genetic clones at a given time point, indicating absence of genetic fixation. In leukemia, where repeated sampling of the malignant cells is possible, early cancer cell clones may ‘go into hiding’, only to reappear in recurrent tumors, defying the logic of the stepwise clonal selection of SMT (“linear evolution”).	[35,37,42–45]
		Such complex clonal dynamics supports the possibility of “multi-clonal” origin of cancer but does not preclude monoclonal origin—the default interpretation of “truncal mutations” as “founder mutations”. Such mutations are just pedigree markers and ignore subclonal non-genetic (but inheritable) pathological cell states as initiating states (see point 8).	[36,46,47]
**7**	Cell lineage tracing at single-cell resolution reveals rather stochastic clonal kinetics of recurrence, and relapsing tumors often have no lineage relationship to the primary tumor, defying genetic bottlenecks as a signature of stepwise clonal selection.	Cell lineage tracking at single-cell resolution (based on genetic markers, DNA bar-coding or immune receptor clonotype) show that recurrence after regression consists of multiple genetic clones, many not seen at diagnosis.	[25,45,48–52]
		By contrast, some recurrent genetic alterations (notably, copy number variations) can arise rapidly and independently in early stage (normal) cells, consistent with punctuated equilibrium evolution (discussed in the main text).	[53]
		Clones in relapsing tumors (after removal of the primary tumor) can be genetically unrelated to the original tumor, thus representing an independent lineage.	
		These findings suggest the existence of tissue fields that establish a proclivity for acquiring particular mutations that drive recurrence.	[54]
8	Existence of multiple ‘clusters’ of cells and the dispersion within each cluster in single-cell transcriptomics of clonal cell populations reveal ubiquitous non-genetic phenotype heterogeneity as a manifestation of phenotypic plasticity, which is a fundamental property of clonal (cancer) cell populations.	Such phenotypic cell-to-cell variability given identical genotype is not just “molecular noise”, but reflects enduring functional diversity with biological consequences (see points 9 and 10):	[55]
		Disparity between genotype and phenotype. Cancer cells carrying oncogenic mutations can have transcriptomes indistinguishable from non-mutated cells (i.e., are intermixed and co-cluster with wild-type cells).	[49,50,56]
		State transitions between transcriptomic cell clusters (subpopulations). Bar-coding combined with single-cell transcriptomics in perturbation or repopulation experiments expose intrinsic and externally regulated dynamics with mass transitions (flows) of cells between cell clusters.	[57, 58]
		Progenitor cells containing oncogenic mutations can still differentiate and contribute to various mature lineages—albeit possibly with altered transition rates.	[59]
		Distinct subpopulations in isogenic cell populations display distinct responses to perturbations. Cells in different subclusters respond differently to growth factors or drugs and even have distinct susceptibility to oncogenic mutations.	[60]
9	Alternative isogenic cell states can represent ‘persister’ cells that may confer tolerance to treatment, and could be selected for; yet, they are also reversible as the changes are non-genetic.	Persister cells represent a specific form of non-genetic phenotypic plasticity (see point 8). Since they endure over multiple cell generations, they can serve as a substrate for Darwinian selection without need for genetic mutations. The reversibility of the persister state implies that the relapsed treatment-refractory tumor can become sensitive again to the same drug after a certain period without treatment, defying the idea of SMT. Entry into persister states can be spontaneous/stochastic or deterministically induced by a signal (see point 10).	[61] [55,62–64]
10	Alternative isogenic cell states that confer therapy resistance (including persister states) can be *induced* and are not necessarily “selected for” by treatment (point 9), imparting some Lamarckian (as opposed to Darwinian) dynamics to tumor progression.	The appearance of stem-like, treatment resistant cells is an active, regulated cell response to treatment stress, mediated by specific signaling pathways, and not a passive selection of pre-existing mutants. The stress-induced stemness/resistance can be cell-autonomous or non-autonomous, triggered by signals from other tumor cells or the microenvironment, such as Wnt and TGFβ. Non-genetic induced resistance may have evolved as a rapid survival response following tissue injury, ‘buying time’ for cells to accumulate mutations via natural selection of mutant clones that are resistant.	[65–76] [55,77–81].

Large-scale cancer genome sequencing endeavors, such as The Cancer Genome Atlas (TCGA) [82], launched in 2006 by the U.S. National Institutes of Health, have uncovered a mesmerizing breadth of genetic mutations associated with cancer. The vast, nearly chaotic diversity of genetic alterations found within the same nominal tumor type between patients is staggering and questions a deterministic logic of causation of cancer by mutations in oncogenic pathways (Table 1). However, patterns, such as recurrent mutational signatures and chromosomal rearrangements, are characteristic for distinct tumor types and stages [53,83] and point to a complex interplay between stochastic elements and some rule-governed biological causation.

The immense diversity of functions of the (mutated) molecular pathways allegedly linked to cancer causation [21], and the fact that cancer is a robust, universal phenomenon amongst metazoans, tells a story of principles of development and evolution of multi-cellular life behind the very existence and the near- inevitability of neoplasia, transcending phylogenesis, ontogenesis and oncogenesis [84–94]. However, in the quest for predictive biomarkers and molecular targets, the cancer research community has abandoned deep thinking for deep sequencing, interpreting data through the lens of clinical translation detached from fundamental biology.

## The core of the genetic paradigm: Somatic Darwinian evolution

To revisit the role of mutations in carcinogenesis, we need to recall a central corollary of the cancer genetics paradigm that links somatic mutations to the cancer phenotype and cancer progression: namely, the idea of somatic Darwinian evolution of the mutated cells. Herein, random mutations may (by chance) confer functional advantages, e.g., promote autonomous proliferation that increase cell “fitness” [95]. Then, under natural selection in the cell population, those cells carrying mutations that confer a “fitter” phenotype would clonally expand, with the “fittest” clone (derived from a single cell) eventually taking over the population. This Neo-Darwinian scheme is applied to tumors in a loose, qualitative, if not figurative manner [96], often departing from rigorous concepts of evolutionary biology, and eventually becoming known as the SMT [97]. The idea of SMT has its origin in 1914 when the embryologist Theodor Boveri proposed that cancer originates from chromatin alterations in a cell. It was later recast in terms of accumulation of a number of genetic mutations by Bauer and Nordling in the 1950s [98, 99], implying a need for increased cellular proliferation that is acquired as a novel trait—but still without invoking Darwinian positive selection.

The concept of carcinogenesis as an explicit process of (somatic) evolution in the Neo-Darwinian sense was popularized by Nowell’s seminal paper in 1976 [2]. However, he introduced a pluralistic view:

*“…this correlation between observable genetic change and tumor progression does not, of course, prove causality, and some workers believe that at least some aspects of the evolutionary process, (…) may represent epigenetic, rather than genetic phenomena.”*

These cautionary words have been largely left out of the equation of cancer genetics in the following 50 years. Despite its rapid ascendance, SMT remains a conjecture supported by spotty, selective evidence rather than a precisely formulated theory, notwithstanding its use of mathematical formalisms [100]. Specifically, SMT left out broader considerations of historicity (ontogenesis and phylogenesis) and physiology. First, autonomous proliferation appears to be a latent ‘default’ state, ‘baked-in’ in organismal existence already in the last universal common ancestor, and is not an evolutionary novelty that is attained through natural selection in the tissue [101,102]. Second, expansion of faster proliferating cell clones is not limited to tumors, as evident in aging normal tissue and chronic diseases where hyperproliferation results in oligo-clonal expansion [103–105]. Yet, convenient for its simplicity, but misleading in its meaning, the Neo-Darwinian principle has become the unquestioned truth in tumor biology which, as Table 1 shows, is responsible for many of the cracks in the genetic paradigm of cancer.

Robust manifestation of natural selection in tumors would be ‘hard selective sweeps’ [106,107]: the complete overtaking of the tumor cell population by a malignant clone carrying the driver mutation (along with linked, but neutral mutations), representing one step in Vogelstein’s multi-step progression model of linear evolution [4,46]. The genetic fixation (as it is called in population genetics, see Table 1, point 6) of the oncogenic allele in the tumor cell population would be required for the logics of precision oncology that seeks to target a mutation to eradicate a tumor. In reality, a tumor contains thousands of genetically distinct, apparently independent cell clones, giving rise to genetic intra-tumor heterogeneity that came as a shock to the precision oncology world [37,42]. This finding led to attempts of adjusting precision oncology by targeting “trunk mutations” shared by every cancer cell and by combination therapy directed against multiple oncogenic mutations—approaches that still adhere to SMT [108].

But, even if a clone appears to expand faster and dominate the tumor, there is little proof that a complex tumor phenotype is sculpted by natural selection for specific oncogenic mutations that produce said ‘adaptive traits’ as Neo-Darwinism postulates. It is likely correct that simple Darwinian evolution is at display in the case of evasion from targeted therapy, notably kinase inhibitor drugs [41], wherein a point mutation in the kinase protein interferes with multiple aspects of kinase activity energetics, as best studied in the oncoprotein CBR-Abl, as it becomes resistant to the highly selective inhibitor imatinib [109]. But, adaptive selection of such molecular phenotype is a far cry from the suggested generation of complex traits that embody the “hallmarks of cancer”[110,111] by natural selection during tumor progression. Thus, SMT suffers from the same shortcoming as Neo-Darwinian evolution [112] in that it only explains distinct localized adaptive traits obviously linked to the selection pressure but not the emergence of complex, higher-order biological functions.

One must also not forget that neutral variants can be enriched without being selected for (Table 1, points 5, 6) because of (random) genetic drift in relatively small cell populations. In finite cell populations the mathematics of random birth-death processes alone accounts for highly skewed allele frequency distributions that defy our numerical intuition (“the rich get richer—without being more capable”) [39,113,114], although neutral drift may be overestimated because of late appearing subclonal driver mutations [115].

Complicating the picture of somatic evolution are interactions at a higher level, like those between subclones of cancer cells that affect fitness of the entire tumor [116–119], creating non-cell autonomous dynamics [104]. Beyond direct mutual stimulation, the distinct clones may functionally complement each other because of their ‘specialist’ capabilities that support the cancer cell community, for instance, through metabolism that alters pH and nutrients in the intercellular space, detoxification, promotion of angiogenesis, alteration of tissue mechanics [105], or averting anti-tumor immunity. These biological functions are not sufficient to grow a tumor themselves. Conversely, some tumor cells may keep their more malignant neighbors at bay and must not be killed [106].

Thus, if we view the tumor as an evolving ecosystem, as has become fashionable, we also must embrace evolutionary/ecological principles that have emerged in the past few decades and that question the dominance of the simple scheme of ‘natural selection’ of Neo-Darwinism [55,120–124]. These include punctuated equilibrium (stasis with sudden bursts of evolutionary events) [125,126], group selection (for fitness of a group due to social behaviors) [126,127], niche construction (evolving organism modifies its own selective environment) [128] and structuralism (natural selection must obey constraints of physics, chemistry and geometry, etc. which contribute to shaping the phenotype with no selection) [129].

Of particular relevance is the group of evolutionary theories that emphasize phenotypic plasticity [123,130,131]. These theories all suggest that the direction of evolutionary adaptation of populations ‘tracks’ the physiological adaptation of the individual to its environment as enabled by regulated phenotype plasticity, such as acclimatization. These theories include genetic assimilation/genomic accommodation [123,130–132], the Baldwin effect and canalization [133,134], and phenotype-first dynamics [94,135]. These mutation-less mechanisms may ‘lubricate’ and channel natural selection; thus, they actually support Darwinian natural selection by making evolution of adaptive traits more likely in view of the ‘unlikely’ mutations that need to happen. Thus, despite being largely absent in SMT literature, all these theories indirectly support Darwinism by considering non-genetic plasticity, thereby relaxing the rigid one-genotype-one-phenotype constraint.

## Overcoming the limitations of the current paradigm: Expand it or replace it?

Calls for overcoming the blinders of the genetic paradigm have a long history. What is new is that, first, the same omics technologies employed to identify new oncogenic mutations have also been used to better understand non-genetic processes in tumorigenesis that involve cell plasticity and tissue organization. And second, the quest for drug targets or biomarkers have meanwhile reduced much of cancer research from a scholarly biological discipline to a transactional operation concerned more with cataloguing molecular alterations and finding associations, than with understanding fundamental principles using formal theory [136]. The zest for intellectual discourse on the nature of cancer, the type of scholarly discourse that advances all scientific disciplines, is long gone, or viewed upon with suspicion.

Since one should not postulate the end of a paradigm without specifying the ‘alternatives’ that may correct or replace it, we will discuss here two sets of ideas that have been sidelined by the dominance of the genetic paradigm of cancer. First, that “cancer is not a disease of the genes”, but of gene regulation and thus, of the cell. We must consider the dynamics of the collective action of genes in a network that governs cell behaviors. Second, that ‘cancer is not a disease of the cell’, but of tissues [137]; we must consider principles of tissue organization. Both of these ‘alternatives’ are discussed in detail below.

To interpret observations afforded by powerful new technologies, we more than ever need a solid framework grounded in biological principles that must be rigorously formulated (but may not yet be mathematically formalized) [138]. Cancer research needs to return from current transactional activities to the scholarly investigations enjoyed by generations of biologists who have placed new findings in the context of organisms, their development, physiology and evolution [136,139]. As Philip Ball points out, the apparent lack of ‘progress’ in the biological sciences may be related to the adoption of a mistaken paradigm [140]. Herein, we will illuminate the paths out of the genetic paradigm, touching on scientific, epistemological and sociological aspects of cancer research.

## History of anomalies before the era of molecular genetics: The normalization of cancer

If so many sequencing results are inconsistent with SMT, why do they not trigger a rethink among cancer researchers? Revisiting an overused idea of the philosopher Thomas Kuhn on how science advances, “anomalies” (findings inconsistent with respect to established knowledge) should have begun a shift long ago in the paradigm of what he calls “normal science” [141]. Anomalies that challenge SMT are not new [28,99,142–145]. Puzzling findings at the organismal level predate the recent wave of hard-to-explain results produced by deep genome sequencing by more than half a century; they question the notion of cancer as a disease of the cell that is irreversibly transformed to a malignant phenotype by genetic alterations. On center stage is the ‘normalization’ of the cancerous cell accomplished by physical contact with a pertinent ‘embryonic field’ [146], which has prompted some researchers to view cancer as a problem of cellular differentiation [146–148].

 “Normalization” is perhaps the most prosaic anomaly to the mutation paradigm. When Peter Nowell popularized clonal evolution of cancer cells [2], Beatrice Mintz’s group presented evidence that teratocarcinoma cells injected into mouse embryos (blastocysts) gave rise to cancer-free chimeric mice in which the mutated cancer cells were present in most organs. Such reversion of the malignant phenotype (i.e., normalization) within a proper tissue context has been reproduced in a variety of animal models [149]. Normalization is also observed during the ‘maturation’ of human tumors, whereby proliferating tumor cells sometimes develop into mature, non-proliferating cells, as most clearly seen in neuroblastoma, where cancer cells become ganglion cells [150,151]. In addition, some therapies, including target-selective inhibition of oncogenes aimed at killing cells, often trigger a wave of differentiation of the immature (mutated) cancer cells into specialized postmitotic cells, despite not being designed as differentiation therapy. This is most dramatically apparent in “differentiation syndrome”, the dreaded surge of differentiated neutrophils in the treatment of acute myeloid leukemia [152].

Thus, a latent developmental potential of cancer cells is occasionally realized, allowing the cells to (non-genetically) enter a mature state, provided a biological context that opens access to trajectories of physiological development. The old notion of cancer as a differentiation arrest is consistent with the potential for normalization of the malignant phenotype when the appropriate signals relieve the arrest [27,153–157]. Conversely, stressors, notably cytotoxic treatment itself, often cause ‘de-differentiation’ in the cells that survive treatment. As noted in point 10 of Table 1, cell stress can, independent of mutations, induce a stem-like state that confers xenobiotic resistance, reorganize the tumor microenvironment (TME), and promote a (pathological) healing by exploiting developmental fields, all in the absence of Darwinian selection of genetic mutants [158]. The logical inverse of Mintz’s experiments is the work that produced the teratocarcinoma cells used in those very experiments; these cells were generated by implanting normal embryos under the testicular capsule [159]. Later, it was observed that grafting normal (presumably mutation-free) embryonic stem cells into the mouse can produce teratocarcinoma [138].

These historical examples show that both differentiation (or ‘normalization’ if it happens in cancer cells) and de-differentiation (which creates the cancerous stem-like phenotype) are biological processes intimately linked to the existence of cancer. They represent drastic phenotype conversions that lack the characteristic of selective clonal expansion of mutated cells as the agent of pathological change. Instead, phenotypic transitions are controlled by a complex tissue context, which implies the involvement of an array of often poorly understood, coordinated signals.

## The cell phenotype as collective actions of genes

A new malignant cell phenotype in tumor progression is still often explained by a genetic mutation that follows the conventional ‘one gene—one trait’ scheme. But, as widely noted, there is no simple correspondence (i.e., no 1:1 mapping) between genotype and phenotype [160]; genes directly code for proteins, not phenotypes, and they do not act alone. Gene loci interact with one another, via regulatory proteins that they encode, to collectively produce a cell phenotype [161,162]. The fundamental principle behind the fact that cells within an isogenic (clonal) cell population can have distinct phenotypes, as manifested in the existence of the cell types of the metazoan body, is that cells differ not in their genomes but in their genome-wide configuration of the activities of all gene loci. Such gene activity configurations produce the distinct gene expression patterns or transcriptomes that we can measure and interpret as the molecular basis of cell phenotypes.

**Fig 1:**
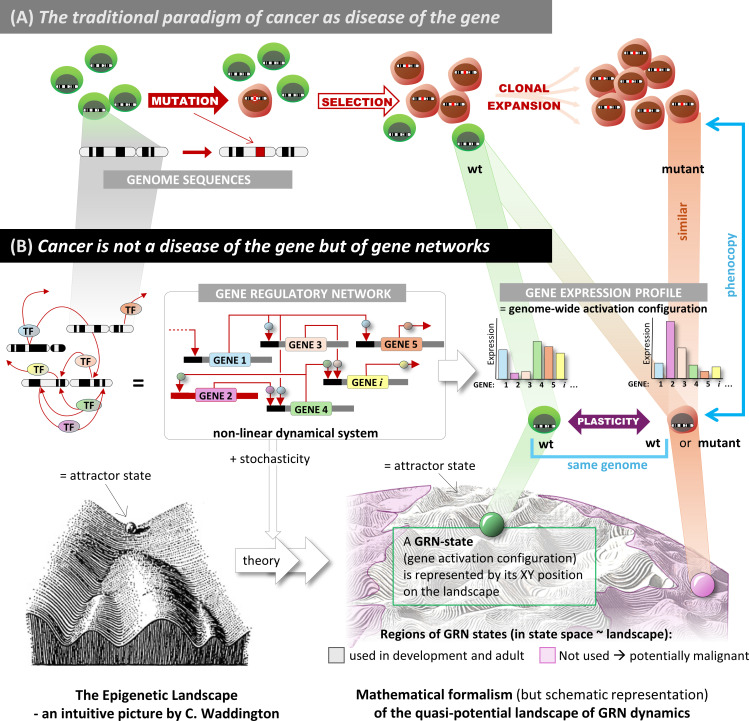
The paradigm of cancer as a disease of gene regulation. (A) The current paradigm of genetic mutation as the source of phenotypic change that drives tumorigenesis. (B) The new paradigm that explains cell phenotype changes by a concerted change of gene expression: the concept of the gene regulatory network (GRN) with gene-gene interaction mediated by transcription factors (TFs) or regulatory RNAs generalized a “wiring diagram”. It governs the dynamics of gene expression profiles that intuitively (Waddington, left) or with mathematical theory (right) can be represented as a landscape. In the landscape, the attractor states of the GRN, which define stable cell phenotypes, are the valleys. The theory predicts many more attractor states than can be occupied by cells in physiological states (cell types, in gray regions). The unused attractors (red) encode the gene expression profile thought to map into those of cancer cells.

The multiplicity of cell phenotypes given one same genome obviously defies the tacit assumption of a bijective mapping between genotype and phenotype. Such a relationship is required for Neo-Darwinian logic to work: natural selection enriches a population for a given genotype via a corresponding phenotype to which a fixed ‘fitness’ value is assigned that determines the selection advantage [55,112]. The phenotype diversification that produces the adult cell types from an unchanging genome follows the rules of development that are imposed by the regulatory interactions between the gene loci (and by interactions between the various cells in the tissue). The interactions between the genes are hard-wired in the genomic sequence and collectively form a complex network, the gene regulatory network (GRN), that orchestrates the activities of genes to produce those gene expression patterns that underlie the biologically meaningful phenotypes (Fig 1). Such a “genetic network” was first proposed by Jacob and Monod in the 1960s after they discovered that genes regulate other genes [163]. The central idea, as can be formulated mathematically, is that in coordinating the activities of genes, the GRN tends to interlock them in distinct, self-stabilizing gene expression configurations, called attractor states (valleys in Fig 1B). A key postulate, formulated in 1969 by Stuart Kauffman, is that attractors represent the gene expression patterns that correspond to cell types (or functional cell states) [162,164–166]. Being attractor states, such gene expression patterns are robust to perturbations; they re-establish spontaneously after disturbances, and thus can be inherited across cell generations. The existence of multiple attractor states is a feature of a particular class of complex dynamical systems to which GRNs belong [165].

Gene regulatory interactions of a given genome are implemented by transcription factors (TFs), and also by regulatory RNAs and chromosomal configurations. TF proteins and regulatory RNAs bind their targets in a sequence-specific manner. Thus, their regulatory action is highly gene locus-specific and is written in the genome; they collectively define the “wiring diagram” of the GRN (i.e., which gene regulates which genes, and how and under what conditions). The GRN was wired by evolution, e.g., via tuning of TF DNA binding motif sequences (*trans-*regulation) and their cognate target elements (*cis-*regulation). This resulted in binding specificities between regulators; such gene expression across all loci was forced into a set of genome-wide patterns, allowing the network to produce attractors (stable patterns) that encode meaningful cell phenotypes. The locus-specific TFs and regulatory RNAs are supported by the non-locus-specific apparatus of epigenetic modification (covalent marks on DNA and histone proteins) that sharpen signals and modulate temporal patterns [167]. Thus, the GRN is a distributed information processing system of mutually regulating genes that form a web of feedback loops—contrary to early views of genetic networks as hierarchical cascades of causation that rigidly link genotype to phenotype [168].

**Fig 2:**
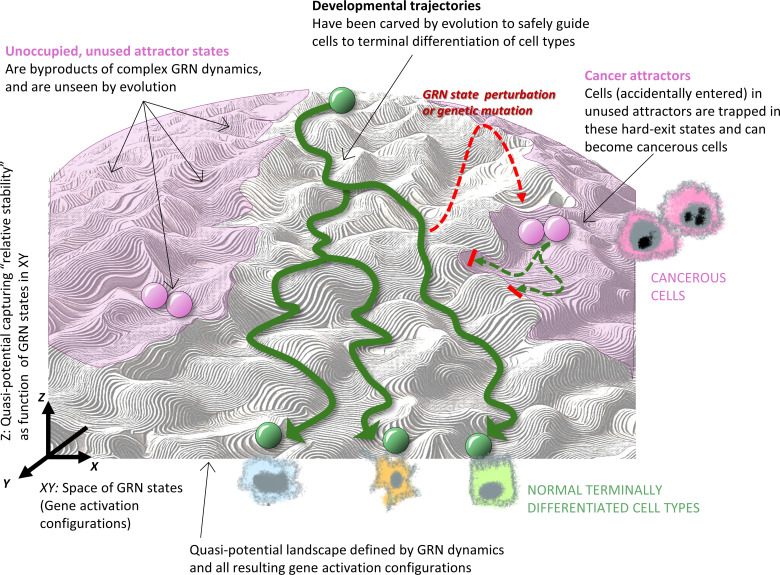
Cancer attractors on the epigenetic landscape. Mathematically, the epigenetic landscape can be extended to regions that represent gene activation configurations (in pink) that exist in theory but are never realized during development and in physiological tissues (green traces descending the hill in the gray regions). The unused regions (pink) contain unoccupied attractors—the cancer attractors. Under abnormal conditions cells can, upon massive perturbation of their gene expression profile, enter such cancer attractors that lack a path to the normal mature cell type attractors (bottom)—and thus are “maturation-arrested” and, if associated gene activation configurations are compatible with cell viability, can become cancerous. Mutations rewire the gene regulatory network (GRN), thus altering the dynamics, which can result in lowering of the barriers (hills) that prevent cells from leaving the physiological regions, facilitating the occupation of cancer attractors.

The GRN is not a clockwork. Stochasticity in cell-type diversification had already been proposed by Kupiec in 1983 [161]. Thermal fluctuations of molecule concentrations (due to the small number of molecules in cells) add a layer of non-determinism to the rules of the GRN, allowing attractor states to be represented as probabilities of being occupied [169–171]. This coexistence of deterministic logic and its probabilistic realization permits the definition of a potential-like quantity, which is aptly represented by Waddington’s epigenetic landscape, in which valleys correspond to attractor states and whose depth represent the relative stability of the cell phenotypes that they encode (Fig 2) [172,173]. (For simplicity, but sufficient for this discussion, we deal here only with “fixed-point attractors” and omit more complex dynamical structures, such as “limit cycles”, in which cells are trapped into a closed loop trajectory, such as the cell division cycle [174,175].)

Now, here is a crucial corollary: on the epigenetic landscape of a given GRN, there exist, for mathematical and network-evolution reasons, many more attractor states than are occupied by cells in the healthy adult organism [176,177]. The phenotype of these unused attractors has been proposed to represent malignant cells [89,178]; in this view, cancer is a possibility immanent to metazoans, and hence is less suited to be seen as “being caused by something”, but rather is primarily the “unleashing of something latent”. Carcinogenesis would then be the accidental entry into these unused latently present attractors, which thus have been referred to as “cancer attractors” [178]. And these must be avoided by normal cells in development and under physiological conditions (Fig 2). This is the reason why the old adage of the molecular biologist that development is “tightly regulated” is more profound than one may think.

## Waddington’s epigenetic landscape and cancer attractors

In the modern interpretation of the epigenetic landscape, cancer is thus a built-in but normally not realized feature, as epitomized by the unused attractors. Since, in physiological conditions, these attractors are unoccupied, they are not exposed as phenotypes and thus, they are ’not seen’ by natural selection; most of these states would establish gene expression patterns incompatible with life in the modern metazoan tissue. Those few unused attractors that can sustain viable cells in the tissue are not fine-tuned by selection. When occupied by cells that thus, by definition, become neoplastic, cancer attractors would command phenotypes with primordial behaviors not shaped by metazoan evolution to support cell societies that maintain tissue homeostasis [91,179]. In this perspective, cancer would represent atavism (“reversion to an ancestral form”): cancer cells would revive “selfish” protozoan functionalities, however imperfect [88,90,180,181]. More generally speaking, unused attractors represent cell “programs” of the phylogenetic past [182]. The accidental yet highly organized nature of vestigial phenotypes and their consistent reoccurrence is naturally explained by re-occupation of unused attractors that are an integral part of the same epigenetic landscape that is encoded by the genome and governs the unfolding of the normal extant phenotype. (The cancer atavism idea should, in its argumentation logic, not be conflated with the notion of proliferation and motility being the “default state” of cells (discussed in detail later) [91,179,183].)

Cancer cells are also stuck in an ontogenetic past. Because unused attractors do not partake in development of later evolved mature tissues, they are more likely to produce developmentally immature, dysfunctional traits that lack fine-tuned efficient homeostasis (mathematically represented by deep, smooth basins of attraction). For instance, being ‘unevolved’, they are likely to lack the sophisticated metazoan capacities to contribute to tissue morphogenesis or to replicate genomes with absolute fidelity. Most importantly, they are not connected by developmental paths to the mature cells’ attractor states (Fig 2, right). Such paths were carved by evolution (via GRN wiring) into the epigenetic landscape to ensure that normal immature cells respond to appropriate tissue signals during development and efficiently differentiate, while being shielded from undue entry into the unevolved cancer attractors (green curved arrows in Fig 2). Once trapped in such attractors of imperfect homeostasis, cells are easily perturbed by hypoxic, inflamed and or otherwise stressed stroma [184], all of which are known to cause genome and epigenomic instability, resulting in genetic alterations, further promoted by the absence of optimized DNA repair systems [185,186]. The immaturity of these cells places them closer to early developmental stages, in line with their expression of embryonic cell traits, such as accelerated cell division, multi-lineage potency, tolerance to genomic instability and xenobiotics—all features shared by cancer cells.

Having scant access to the physiological attractors of differentiated cell state [89] (Fig 2), cells in cancer attractors are also incapable of differentiation, hence the pathologists’ notion of maturation arrest [148]. The ‘accidental’ entry into cancer attractors does not depend on a specific mutation; instead, all kinds on non-genetic stressors that produce particular non-physiological, unstable gene expression patterns, amplified by stochastic fluctuations of transcriptomes, can trigger exit from normal cell type attractors and entry into a nearby cancer attractor (see point 10, Table 1).

## Rethinking the concept of genetic causality for cancer

The concept of accidental entry of cells into a latently existing but undesired attractor and then being trapped in it, supports the notion of cancer as a process of unspecific “unleashing” (of a potential) rather than of specific “de novo causation”. Cancer attractors explain the unfathomable diversity of molecular disruptions and perturbations that all can accidentally trigger the entry into them and produce, as if orchestrated by an invisible hand, a specific gene activation configuration that governs the cancerous phenotype. These disruptions can result from an immense diversity of random genetic alterations that affect proteins of a variety of functional classes [21] or from a vast array of defects in regulatory interactions, from the rewiring of gene–gene interactions by genomic translocations (including creating novel fusion proteins or swapping promoters), the dysregulation of non-coding RNAs and RNA splicing, to altered function of broadly acting epigenetic and chromatin-modifying regulators. The immense diversity of mechanisms, all accidental, that can converge to producing the cancerous phenotype with minimal help by Darwinian selection, is perhaps the most prosaic yet profound manifestation of cancer attractors as latent structures in dark regions of the vast state space of gene activation patterns.

So, if genetic alterations are involved, what is our criticism of the claim of a genetic cause of cancer in the current paradigm? Cancer cannot be explained by explicit genetic causation in the traditional sense of “a gene X for that phenotype Y” being defective, as is readily achieved for mendelian diseases. Instead, we need to consider the collective action of genes, which we now can formalize with the epigenetic landscape, as well as cell population dynamics and tissue context. The landscape provides a formalism that places the role of a genetic anomaly for tumorigenesis in a new light: genetic mutations essentially alter the GRN wiring. In mathematical models that map the wiring diagram to the landscape topography, a localized GRN change (a point mutation or a larger scale rearrangement) is a localized rewiring and will most often just gently distort the topography of the landscape. There are only so many ways a landscape can gently change its shape (while still ensuring viability)—and one typical way is by altering the barrier height that separate the attractors. Such a distortion in turn affects the cell trajectories. For instance, it could facilitate the accidental entry into a cancer attractor from a given place in the normal trajectory, driven by molecular noise or non-mutagenic environmental stress (details in [55,158]). It is in this convoluted sense that one can admit that genetic mutations can “cause” cancer.

Despite the absence of developmental paths to normal mature states, the epigenetic landscape still grants cells in cancer attractors an intrinsic potential for normalization, as manifested in the rare reversion of the cancerous phenotype under particular experimental conditions [153,155,156,187,188]. The reversion potential is rarely realized because very distinct tissue constellations are required to overcome the energy barrier to exit the cancer attractor, as seen in the particular instance of neuroblastoma that can spontaneously normalize. The difficulty of normalization is consistent with the limited success of differentiation therapy [189,190] and with the exceeding rarity of “spontaneous remission” of cancer despite the theoretical reversibility of cell states [191].

We can now more precisely articulate an indirect, convoluted role of genetic mutations in cancer to reconcile with the SMT view. Both the genetic paradigm of cancer and Neo-Darwinism rest on the tacit assumption of a 1:1 mapping between genotype and phenotype [192]. In this view, one must, by logical necessity, explain any phenotypic change by a genetic mutation. But, as computer simulations have suggested, most mutations in the core GRN (which in principle alter the wiring of the GRN) do not qualitatively affect the topography of the epigenetic landscape (e.g., they typically do not destroy or create attractor states), but as said above, cause a gentle distortion. Thus, the majority of mutations, even those that alter gene activity, are readily buffered away. However, mild distortions due to GRN rewiring can alter the relative stabilities of attractors, and thus may affect transition rates between attractors (e.g., stem cell and differentiated state and senescent states), including rates for epithelial-mesenchymal transitions (EMT)[193,194] and entry to the unused attractors [195,196]. Altered transition rates in turn lead to imbalances in tissue homeostasis. One manifestation is the widely observed *trans-*differentiation event (lineage switching, “lineage infidelity”) seen in malignancy.

As to the much-needed higher-level organismal biology view of cancer as a complex disease, a first step towards organicism is in the interpretation of large-scale genomic alterations as the source of cell physiological and not genetic informational departure from the norm. Aneuploidy and polyploidy in cancer cells [197] often result in the formation of polyploid giant cancer cells that are found in nearly half of all high-grade cancers [198]. Polyploidy and associated expression of meiotic genes in malignant cells offer a link between cancer biology and organismal biology at a larger timescale and could represent a rather generic alternative attractor that is often accessed by tumorigenic dysregulation of the GRN. In fact, since tumors often reactivate diapause and gametogenesis programs that mark the entry into a new sexual reproduction cycle and epitomize the immortality of the germline, it has been hypothesized that cancer may recapitulate, albeit in an aberrant and abortive manner, the life cycle of organisms [180,199,200].

Finally, beyond its putative role in generating mutations (and neoantigens) [201], there are also organismal consequences of genomic instability (the “mutator phenotype”), which itself is more likely a consequence of the abnormal phenotype produced by unevolved attractors than the needed source of cancer-causing mutations. Indeed, the idea of what actually “came first” was articulated by Richmond Prehn: *“…it may be more correct to say that cancers beget mutations than it is to say that mutations beget cancers*” [202]. Then, we shall also remember the physical, not informational consequences of genome instability: the DNA repair response triggered by genomic instabilities produces unphysiological DNA configurations (breakpoints, stalled replication forks, RNA–DNA hybrids, double- and single-stranded DNA, etc.) that leak into the cytoplasm, which in turn, mistaken for viral invasion, activates stress and inflammatory responses via the NFkB and STING/interferon pathways, thus affecting the cell’s microenvironment [203,204]. This brings us to the tissue level processes.

## Cancer as a tissue-based disease: The tissue organization field theory of carcinogenesis

The interpretation of genome sequence-based lineage reconstruction (Table 1, point 6) defaults to the view that the tumor originates from a single cell, the “initiating” or “founder”, cancer cell. This is correct in a fundamental, almost trivial way because a group of cells always has a common ancestor—it just depends on how far back one traces the pedigree (up to the zygote). The idea of one initiating cell in which the truncal (“tumor initiating”) mutation in the clonal tree happened, making it a founder cell [36], ignores that its outgrowth has to depend on its neighboring cells (normal or transformed) that provide the critical tissue context for it to survive and also ignores the multiplicity of distinct cell phenotypes within an isogenic clone. Conversely, not all cells that contain that same early mutation (that would allow for later oncogenic, otherwise lethal mutations to accumulate) will become cancerous—see Table 1, points 6 and 7 [108].

**Fig 3:**
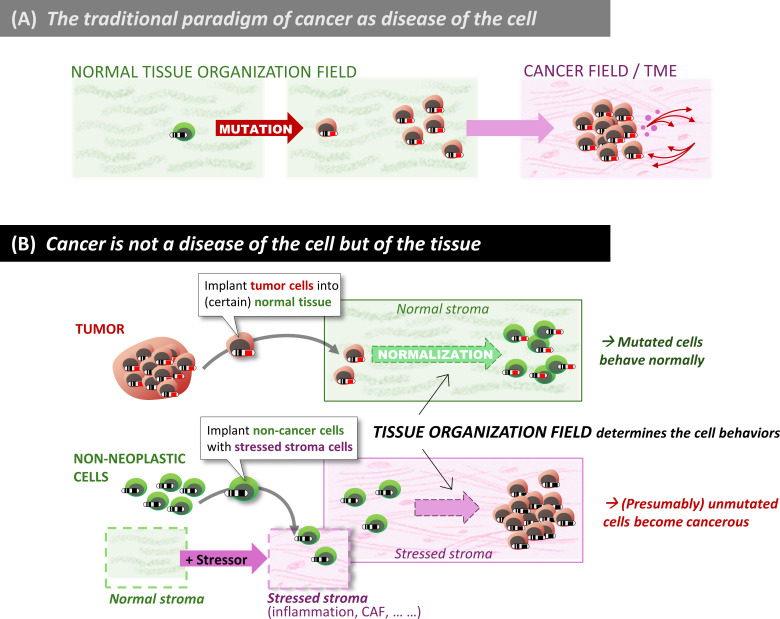
Cancer as a disease of the tissue, not the cell. (A) The paradigm of abnormal cells as the source of tumors. (B) The new paradigm of cancer as a disease of the tissue, explained by schematics of seminal experiments that establish the phenomenon of normalization of cancerous cells by the stroma, as well as the converse, i.e., the initiation of tumor from “healthy cells” due to carcinogenic stressors acting on the stroma. Note that this second paradigm shift implicitly requires non-genetic plasticity of cancer cells, as proposed in the first paradigm shift (Fig 1). In the traditional paradigm, plasticity is assumed only for the (non-transformed) stromal cells.

Empirical evidence contradicting the view of “cancer as a disease of the cell” was already available over 60 years ago, when David W. Smithers published an essay entitled “An Attack to Cytologism” [205]. Smithers refuted the idea that cancer originates from an abnormal cell. His and others’ observations suggested that cancer is not a disease that starts from a single tumor-initiating cell, but rather an anomaly of tissue organization (Fig 3). (This argument only proposes that it is not as simple as postulating “a” cancer cell but does not deny the existence of individual cells with enhanced potential to drive tumor growth).

A compelling Illustration of the need for a tissue-based rather than cell-centric view is the finding that while a bulk injection of hepatocarcinoma cells into the liver generates a liver tumor, injection of the same amounts of the same cell type into the spleen, which distributes transplanted cells individually throughout the liver, fails to generate a tumor [153]. Importantly, the cells injected into the spleen became normalized in the liver by direct contact with their neighboring normal hepatic cells and were incorporated into the liver’s normal structure, underscoring the role of non-genetic, environment-regulated cell phenotype plasticity at the demarcation between normalcy and neoplasia [154,206]. These observations led to the concept that there is no “cancer cell” [101] because cells declared as such (e.g., because of canonical oncogenic mutations) behave as normal cells (Table 1, Point 2).

Instead, cancer can be broadly understood as “development gone awry”. Within this perspective, the tissue organization field theory is based on two principles that unite phylogenesis and ontogenesis. Firstly, the default state of all cells is constitutive proliferation with variation and motility. Therefore, proliferation and motility do not require an explanation; instead, what ought to be explained is why cells do not proliferate and move. Secondly, cancer is a tissue-based disease, whereby the tissue organization constraints to the default state of its cells are weakened. Consequently, cells become freed to express their default state, thus proliferating, generating variation and moving. This explains tumor growth by accrual of new cells, as well as invasion and metastasis [91,102].

The other major facet of carcinogenesis as a tissue-level phenomenon pertains to the role of ‘non-cancerous’ cells in the TME or the stroma. The idea that the TME, consisting of extracellular matrix (ECM), mesenchymal cells, and resident and invading immune cells, plays a role in carcinogenesis was accepted by the cancer community only in the 1990s, despite pointed reference by the pathologist Rudolph Virchow more than 150 years ago [207]. The all too obvious role of the TME in carcinogenesis, which involves angiogenesis, inflammation, immune suppression and cancer-associated fibroblasts (CAFs) [208], soon led to exploding research funding in this area. However, the stromal component has mostly been seen as a necessary enabler and rarely as a driver of carcinogenesis. It provided the biological embodiment of the convenient notion of context that is often invoked to mitigate anomalies plaguing the SMT. Throughout the 20th century, carcinogenesis was considered a genetic disease of the cancer cell despite accumulating experimental evidence pointing to a causative role of the stroma [209]. For example, the fact that injection of a chemical carcinogen administered intraperitoneally generates mammary cancers in susceptible rat strains has been attributed to mutations caused by the carcinogen in the glandular epithelial cells, such as Ha-ras-1 (details below) [210]. However, all cells of the organism are exposed to the carcinogen. From a developmental biology perspective, the question is, which is the target of the carcinogen: the epithelium, the stroma or both?

## Lessons from organogenesis

To understand the relationship between the stroma (the “support” scaffold of an organ) and the parenchyma (the “functional” part of the organ) in cancer, we shall review the process of organogenesis [211,212]. The genesis of tissues and organs entails complex reciprocal interactions between the components of the morphogenetic field defined by a distinct 3D array of cells that gives rise to an autonomous tissue structure or organ [213]. Numerous epithelium/mesenchyme recombination experiments have shown that it is the mesenchyme that determines the type of skin adnexa; for example, wing mesenchyme dictates the presence of chicken feathers regardless of whether it is recombined with epidermis from foot or wing, and scales appear regardless of the origin of the epidermis if recombined with mesenchyme of the foot [214]. Similarly, the branching pattern of the epithelium of salivary and mammary glands is determined by the stroma of the organ [215].

As in organogenesis, the stroma is equally as important in tumorigenesis. When mammary epithelial cells were exposed in vitro either to the mutagenic carcinogen N-nitrosomethylurea (NMU), or to vehicle before being transplanted into the mammary stroma of rats surgically cleared of epithelium [145,183], carcinogenesis only occurred when the stroma was exposed in vivo to NMU, regardless of whether or not the epithelial cells were exposed to the carcinogen (Fig 3). Mammary epithelial cells exposed in vitro to the carcinogen formed phenotypically normal ducts when injected into unexposed stroma. Mutations in the Ha-ras-1 gene, which was assumed to be the cause of cancerization [210], did not correlate with initiation of neoplasia: not only was it often found in both cleared mammary fat pads of vehicle-treated animals and intact mammary glands of untreated animals, but it was also absent in various carcinomas. Such tissue recombination experiments show that the stroma is a crucial target of the carcinogen, while the epithelium is not, and that in this case, mutation in the Ha-ras-1 gene is neither necessary nor sufficient for neoplastic development [216]. A comparable experiment showed that radiation-induced changes in mammary gland stroma contributed to the neoplastic progression of non-irradiated, quasi-normal (TP53-mutated but non-tumorigenic) mammary epithelial cells [217]. Finally, nontumorigenic human prostatic epithelial cells from benign prostate hyperplasia develops into tumors when recombined with prostate cancer stroma and not with normal stroma [218,219]. Recent stromal-epithelial tissue recombinant culture systems and single-cell transcriptomics have begun to unravel the molecular signals that mediate such stromal tumorigenic effects [220].

Not only can carcinogenesis be initiated by injuring the stroma, but a normal stroma can also act as a gate-keeper of neoplastic development by constraining cells belonging to a neoplasm to behave as the normal cells of the organ that form normal tissue structures [221]. The experimental normalization of mutated cells that were once part of a tumor has been reported for a variety of cancer types, among them melanoma (by the neural crest [222]), hepatocarcinoma (by normal liver [223]), or mammary cancer (by normal mammary gland [27,221,224–226]). A compelling demonstration of a tumor-restraining role of stroma is the case of localized pancreatic cancer, where targeting of (originally considered tumor-promoting) CAFs resulted in tumor progression and invasion [227–229].

Intriguingly, the same chemically inert material can cause tumors in animals when implanted as rods but not when injected as powder of the same material and same mass [230]. This suggests that chronic wounding and/or mechanical stress of the tissue causes carcinogenesis through breakdown of physical tissue architecture in the absence of genotoxicity, something that has long been observed in multiple cancer models, underscoring the importance of tissue fields [231–234]. In the light of the central role of the mesenchyme on the determination of the parenchyma phenotype, it is unsurprising to see the surge of data showing a tumor-promoting effect of altered stroma, most notably, ECM composition, fibroblast phenotype (in the form of CAFs) and tumor-promoting chronic inflammation [209]. While these findings have triggered a massive quest for underlying chemical mediators, one should not forget that tissue organization plays not just a supporting, but a determinant role.

The organicist perspective [138] is based on the interdependency of the organism and its organs. It recognizes a circular causal regimen by closure of constraints that makes parts interdependent [235], wherein these constraints are not only molecules, but also biophysical force [236]. Since such a view encompasses tissues not simply as collectives of cells but as emergent anatomical and functional organized entities, it affords little opportunity for discrete molecular targeting, limiting its appeal to reductionist research programs in search of a molecular ‘silver bullet’.

## Explaining anomalies in cancer biology under the SMT paradigm

A common way of explaining the lack of fit between the SMT and data has been to invoke involvement of biological processes not obviously related to oncogenic mutations, such as cellular metabolism, stromal alterations, bacterial and viral carcinogenesis, immune surveillance, etc. that we have characterized as “compromises”. In such hybridization of explanations, old ideas are kept intact, such as the claim that cancer is a disease of the cell and that a normal cell is rendered cancerous by genetic alterations. Justifications often include epimutations [237] due to altered epigenetic marks (epigenetic reprogramming) or references to the old dualism between initiator (mutagenic) and promoter (non-mutagenic) [238].

The concept of dividing the cause of cancer into the initiator, which is the mutagenic event, and the promoter, a chronic (chemical) stress by typically non-genotoxic agents, has been proposed based on the classical experiments of skin carcinogenesis, first performed a century ago. Mice subjected to chemicals known to be mutagenic will develop skin cancer only after ensuing repeated treatment with a non-genotoxic irritant, such as croton oil or the phorbol ester 12-O-tetra-decanoylphorbol-13-acetate (TPA) [192]. Recent genomic sequence analyses reveal that the initial carcinogen indeed caused genetic alterations, which remain silent until application of TPA at a later time point. After TPA treatment, development of papillomas and carcinomas was observed [239]. The elegance of the concept in its modern reincarnation is that the promoter is now understood to not only exert its effect on the mutated cells via non-genetic phenotype switching, but also on the TME via tumorigenic inflammation. The initiator-promoter model is often used to explain the well-known fact that a large fraction of listed carcinogens is non-mutagenic; they may apparently cause cancer by triggering outgrowth of occult mutated cancerous cells. However, this picture is still too simplistic, exemplifying the aforementioned hybridization of old ideas with new findings. At its core, it still maintains a primary causative role of genetic mutations in a single cancer initiating cell, followed by a sequence of causal steps. The qualitative dichotomy between a mutagenic initiator that creates ’cancer cells’ and the non-genetic, tissue-perturbing promoter that expands them may not be as clear-cut. Indeed, the reverse experiment (first treatment with the promoter followed by the initiator) equally produces tumors [240]. This result refutes the classical model that requires that the mutagenic (alleged) initiator must act first. Instead, the reverse experiment suggests merely a synergism between initiator and promoter, albeit a complex one that must involve tissue memory of non-mutagenic perturbations. The promoter/initiator framework by itself is an interesting model, but further investigation should be conducted through the lens of non-genetic dynamics, tissue organization (including the TME) and organismal biology.

Another aspect that has been invoked to alleviate the discrepancies between genome sequencing results and the SMT is epigenetic reprogramming [241]. A solid, formal explanation for enduring changes in the landscape of cell phenotype would have to consider the dynamics of GRNs because epigenetic marks themselves are not explanatory; they are reversible, and the epigenetic modifiers that place these marks are not locus-specific and must be guided by the GRN to coordinate gene loci activities to produce coherent expression patterns that can be locked-in (attractors) [162,167]. Moreover, the attractor stability of a cell phenotype can be disrupted by drastic changes in its microenvironment that affect a large number of regulatory genes to overcome attractor dynamics. This property is evident in the recombination studies with fetal mesenchyme, underscoring the strong leverage that tissue level interactions and tissue architecture possess in perturbing the GRN at multiple points to trigger attractor destabilization and transitions [231,242,243].

Interestingly, the hybridization of concepts to accommodate anomalies is also present in the extended list of the ‘Hallmarks of cancer’. Indeed, hallmarks only serve as attachment points for tenuous molecular causes, dictated by Dennett’s “greedy” reductionism, namely the recourse by scientists to “underestimate the complexities, trying to skip whole layers or levels of theory in their rush to fasten everything securely and neatly to the foundation” [206]. This attitude became evident in the latest installment of the “Hallmarks of cancer” listing [111] that, instead of biological thinking, uses the existence of mutated genetic pathways to motivate new hallmarks that by necessity are explained by them. Notwithstanding, the two new hallmarks, “Unlocking phenotypic plasticity” and “Non-mutational epigenetic programming”, are non-genetic, with ironic redundancy between them [111].

## Conclusions and outlook

Dealing with a complex world, philosophers of science talk about auxiliary hypotheses, which are readily erected using known facts. These abound because complex systems contain a web of causal interactions, as epitomized by the living organism. They offer an uncountable set of auxiliary explanations that can rescue any existing paradigm and not only renders the falsification of a hypothesis meaningless [244,245] but also its defense by excuse arguments. Put differently, the genetic paradigm of cancer is a vague theory and, as the physicist Richard Feynman stated, vague theories cannot be disproven [246], which is even more true for as complex a processes as carcinogenesis. Therefore, interpretation of empirical results guided by formal theory based on some set of first principles, and undaunted by dogmas, is ever more important in an era of easy data acquisition.

Over the years, many proposals outside the paradigm of cancer as a genetic disease have been offered in view of the accumulating findings pointing to shortcomings of the SMT. Some scientists in the SMT camp—still a minority—now begin to embrace a pluralistic view. For instance, we must recall Mike Stratton’s quote in the opening section of this Essay. After his team failed to find mutational signatures specific to endemic squamous cell carcinoma of the esophagus, he concluded:

“*We have found evidence that chemicals might be able to work in different ways other than directly causing mutations to increase a person’s chances of developing cancer. We will have to rethink our ideas about the way in which some cancers develop. It is a crucial lesson*” [205].

We hope that such “crucial lessons” will not be ignored. Pursuing a rigorous explanation of carcinogenesis entails identification of fundamental biological principles placed within a consistent formal theoretical framework, instead of more terminological acrobatics centered around observed altered molecular pathways. As Darwin pointed out to a friend in 1821,

if one were *“only to observe, not theorise… [one] might as well go into a gravel-pit and count the pebbles and describe their colours. How odd it is that everyone should not see that all observation must be for or against some view, if it is to be of any service”* [247].

Theories do not need to be right; they are a practical tool to conduct research. If Darwin’s pebbles are today’s molecular alterations in tumors, then we must not simply be satisfied with their categorization in a system of ingredients that cause tumors, as illustrated by the proposed hallmarks. We must rather regard the hallmarks merely as manifestations of fundamental principles of living organisms. An epistemic shift towards a biological theory of cancer may still be an uphill battle in the current climate of thought created by the ease of data collection and a culture of research that discourages ’disruptive science’ [248]. Here, we have made an argument for dropping the SMT and its epicycles. We presented new and old but sidelined theoretical alternatives to the SMT that embrace theory and organismal biology and can guide experiments and data interpretation. We expect that the diminishing returns from the ceaselessly growing databases of somatic mutations, the equivalent to Darwin’s gravel pit, may soon reach a pivot point. In addition, we hope that then the adherents of the genetic paradigm of cancer will begin to expand their vista on cancer in a profound way.

## Abbreviations

CAFs: cancer-associated fibroblasts
ECM: extracellular matrix
GRN: gene regulatory network
NMU: N-nitrosomethylurea
SMT: somatic mutation theory
TFs: transcription factors
TME: tumor microenvironment
